# Associations of Dietary Inflammatory Potential with Esophageal Precancerous Lesions and Esophageal Squamous-Cell Cancer: A Cross-Sectional Study

**DOI:** 10.3390/nu15184078

**Published:** 2023-09-21

**Authors:** Jingwen Dong, Min Gao, Lin Li, Xiaoyu Pan, Sheng-Yin Chen, Jun Li, Stephanie A. Smith-Warner, Xiaoguang Li, Hui Wang, Jiali Zheng

**Affiliations:** 1 Department of Epidemiology, Harvard TH Chan School of Public Health, Boston, MA 02115, USA; jingwen_dong@hsph.harvard.edu (J.D.); shengyin_chen@hsph.harvard.edu (S.-Y.C.); swarner@hsph.harvard.edu (S.A.S.-W.); 2 School of Public Health, Capital Medical University, Beijing 100069, China; gaomin@ccmu.edu.cn; 3 Cancer Prevention and Treatment Office, Yanting Cancer Hospital, Mianyang 621600, China; lilin09212023@163.com (L.L.); liji0326@163.com (J.L.); 4 Department of Nutrition, Harvard TH Chan School of Public Health, Boston, MA 02115, USA; xiaoyu_pan@hsph.harvard.edu; 5 Department of Food Safety and Toxicology, School of Public Health, Shanghai Jiao Tong University School of Medicine, Shanghai 200025, China; lixg@shsmu.edu.cn (X.L.); huiwang@shsmu.edu.cn (H.W.); 6 Department of Epidemiology and Biostatistics, Shanghai Jiao Tong University School of Medicine, Shanghai 200025, China

**Keywords:** dietary inflammatory index, esophageal precancerous lesions, esophageal squamous-cell cancer, dysplasia, cross-sectional study, chronic inflammation

## Abstract

Chronic inflammation plays a central role in the progression from esophageal precancerous lesions (EPLs) to esophageal squamous-cell cancer (ESCC). However, few studies have investigated the relationship between the overall inflammatory potential of diets and EPLs and ESCC. We aimed to study the association between the Dietary Inflammatory Index (DII) and EPLs and ESCC. As part of the National Cohort of Esophageal Cancer (NCEC) in China, 3967 residents (1993 men and 1974 women) aged from 40 to 69 years living in Yanting County received free gastroscopy screenings from 2017 to 2019. Dietary intake during the past year was assessed at enrollment of the cohort before screening and DII scores were calculated based on 28 food parameters. EPLs (classified into mild, moderate, and severe dysplasia) and ESCC were histologically confirmed by biopsy. Multivariable logistic regression was used to examine the associations of DII scores with EPLs and ESCC. A total of 312 participants were diagnosed with EPLs (226 with mild dysplasia, 40 with moderate dysplasia, and 46 with severe dysplasia) and 72 were diagnosed with ESCC. A statistically significant positive association was observed between DII scores and overall EPLs (OR_T3 vs. T1_ = 1.45, 95%CI = 1.01–2.09); the association was similar but not statistically significant for mild dysplasia (OR_one-unit-increment_ = 1.11, 95%CI = 0.95–1.34) and for moderate and severe dysplasia combined (OR_one-unit-increment_ = 1.15, 95%CI = 0.87–1.51). The association with ESCC was similar in magnitude but not significant, likely due to the small number of cases. In this cross-sectional study of a population in China at high risk of ESCC, DII scores were positively associated with odds of EPLs and ESCC. Consumption of anti-inflammatory foods may be beneficial to prevent EPLs and ESCC.

## 1. Introduction

Esophageal cancer (EC) is a deadly cancer which ranks as the sixth leading cause of cancer-related deaths in the world [1]. There are two major histological subtypes of EC, esophageal adenocarcinoma (EADC) and esophageal squamous-cell carcinoma (ESCC), which have distinct patterns of population distributions and risk factors [2,3]. While EADC is the predominant type of EC in Western countries, ESCC dominates and comprises 90% of all EC cases worldwide. China accounts for nearly half of the ESCC burden globally [2,4,5,6]. In China, more than 90% of EC cases are ESCC [7]. Over 90% of patients with ESCC are diagnosed at middle or late stages, when there are few effective treatments. However, esophageal squamous dysplasia (ESD), the histologic criterion for defining esophageal precancerous lesions (EPLs) that predicts the development of subsequent ESCC, has a much better prognosis, with a 5-year survival rate exceeding 90% [6]. Therefore, identifying modifiable risk factors for EPLs and ESCC is critical to reduce the incidence and mortality of ESCC, especially among high-risk populations in China where ESCC rates are among the highest in the world [8]. In the progression from EPLs to ESCC, chronic inflammation plays a critical role as a substrate for mechanisms which may involve dysfunctions of the immune system and metabolism as well as genetic mutations to promote ESCC development [9,10]. Some dietary factors have been shown to have an impact on both chronic inflammation [11] and ESCC development [2,12]. A literature-derived Dietary Inflammatory Index (DII) was developed to assess dietary inflammatory potential [13]. The DII has been validated among diverse populations by various circulating inflammatory markers including *C*-reactive protein [14] and IL-6 [15], and has been confirmed in the Chinese population [16]. Several case–control studies have shown that the DII was positively linked with the risk of ESCC [17,18,19,20]. However, few studies have investigated the relationships between the DII and EPLs overall and by subgroups with different severity (mild, moderate, and severe dysplasia).

Therefore, we aimed to use the baseline data of the National Cohort of Esophageal Cancer-Prospective Cohort Study of Esophageal Cancer and Precancerous Lesions based on High-Risk Population in China (NCEC-HRP) to investigate the associations of dietary inflammatory potential as assessed with DII scores with EPLs (including subgroups of different severities) and ESCC, and further investigate whether the associations were modified by important lifestyle and demographic factors [21].

## 2. Method

### 2.1. Study Design and Participants

This cross-sectional study used the baseline data of the NCEC-HRP, which were collected from 2017 to 2019. The NCEC-HRP was a multi-center prospective cohort study; the study population and study design have been described in detail previously [21]. Briefly, the NCEC-HRP was established to assess the effect of endoscopic screening on reducing the incidence and mortality of upper gastrointestinal cancer in some high-risk areas in China [21]. The NCEC-HRP study was conducted in eight study sites in different provinces of China. Our study used baseline data from Yanting County, Mianyang City, Sichuan Province, where ESCC incidence and mortality rates rank among the highest in the world [22]. Men and women aged 40–69 years who were permanent residents of six towns (Linnong, Gaodeng, Jinji, Xize, Lianghe, and Fuyi) in Yanting County and selected based on random cluster sampling were invited to participate in the cohort. Residents with a history of cancer, mental disorders, or contraindications for an upper gastrointestinal endoscopic examination and those who were unable to provide informed consent were excluded from enrollment [21,22,23]. Participants underwent a face-to-face interview with well-trained medical staff in Yanting Cancer Hospital. Each participant received an upper gastrointestinal endoscopic examination to screen for upper gastrointestinal cancers [21]. Demographic, health-related, dietary, and other lifestyle factor information was collected using a structured and computer-based questionnaire at enrollment before the upper gastrointestinal endoscopic examination to reduce potential recall bias [24]. A total of 4968 participants were initially enrolled in the cohort and 4252 participants completed the baseline questionnaire and upper endoscopy (response rate of 85.59%) from 2017 to 2019. Written informed consent was obtained from each study participant. This study was approved by the ethics committee of the Cancer Hospital, Chinese Academy of Medical Sciences, in accordance with the Helsinki Declaration.

### 2.2. DII Score Calculation

Dietary intake in the past year before the baseline interview was assessed via a quantitative food frequency questionnaire (FFQ) developed based on a standardized questionnaire designed by the National Cancer Institute but modified based on local foods and dietary habits of rural residents living in southwest China [25]. Participants were asked about their amount of consumption of 76 food items in 21 food groups, including wheat products, rice, other staple foods (millet, corn, sweet potato, etc.), meat, poultry, fish or seafood, eggs, fresh fruits, fresh vegetables, legumes, scallions, ginger and garlic, salted vegetables, pickled vegetables, milk, yogurt, other dairy products, soymilk, nuts, fresh fruit or vegetable juice, carbonated soft drinks, and other soft drinks [26]. Frequency of intake was measured for each food group with the following question: “How many times do you eat every day?” A 5-point response scale (everyday, 4–6 times/week, 1–3 times/week, 1–3 times/month, and almost never) was available for selection. The amount of food consumption was assessed by the question “How many grams or standard portion sizes do you eat each time?” Subsequently, daily food consumption was calculated by multiplying the intake frequency of each food item by the daily intake amount consumed by each individual. Nutrient intake including energy intake was generated by multiplying the food quantity by its nutrient composition taken from the 2019 China Food Composition Table [27]. This FFQ was validated against three 24 h dietary recalls via dietary pattern methods, which suggested a reasonable validity (Spearman’s correlation coefficients: 0.40–0.68) [25].

DII scores were obtained by linking daily dietary intake derived from the FFQ with the inflammatory effect scores of food parameters included in the DII [13]. The design and development of the DII has been published elsewhere [13]. Briefly, inflammatory effect scores were derived for 45 food parameters (i.e., the components of DII) based on the reported effects of various dietary factors on six important inflammatory biomarkers (including interleukin (IL)-1β, IL-6, IL-4 IL-10, *C*-reactive protein (CRP) and tumor necrosis factor (TNF)-α) from ~2000 research articles published up to 2010. In this study, 28 out of the 45 food parameters were used to calculate DII scores, including total fat, energy, protein, carbohydrates, cholesterol, fiber, vitamin A, β-carotene, riboflavin, thiamin, niacin, vitamin E, vitamin C, magnesium, selenium, iron, zinc, folic acid, monounsaturated fatty acids (MUFAs), polyunsaturated fatty acids (PUFAs), vitamin B6, saturated fatty acid, vitamin B12, green/black tea, isoflavones, anthocyanidins, garlic, and onion.

The daily nutrient and food consumption data calculated based on the FFQ were first standardized to a worldwide dietary database, which included the means and standard deviations of the intake of each parameter in the DII score from 11 populations living in different locations globally to create a z-score for each food parameter. The z-score was converted to a percentile value in a normal distribution. Then, the standardized percentile was multiplied by the inflammatory effect score for each DII component. We obtained the overall DII score for each participant after summing the products across all the available DII components [13]. Higher DII scores indicate diets with more pro-inflammatory potential and lower (i.e., more negative) DII scores indicate diets with more anti-inflammatory potential. The DII score has been construct-validated against inflammatory biomarkers in over 40 populations and has been found to be related with higher concentrations of IL-6, high-sensitivity CRP, and TNF-a receptor 2 [14,28].

### 2.3. Covariate Assessment

At enrollment, each participant reported their socio-demographic characteristics, including age (years), sex, marital status (married, not married), educational level (primary school and below, junior school, high school and above), occupation (farmer, non-farmer), household income (<CNY 50,000/year, ≥CNY 50,000/year), as well as lifestyle factors, including smoking status (never, former, and current), alcohol drinking status (never, ever), frequency of physical activity (never or seldom, more than once a week), frequency of eating hot food in the past year (≤1–3 days/month, ≥1–3 days/week), texture of staple food (hard, soft or liquid), and habitual eating speed (slow, normal, fast). Physical activity referred to occasions on which an individual’s heart rate obviously increased due to participation in an activity. Participants also self-rated their health status (good, fair, poor and very poor). History of digestive diseases, including superficial gastritis, atrophic gastritis, hypertrophic gastritis, stomach ulcer, and duodenal ulcer, was reported as “yes” or “no” and a summary variable was derived representing a history of any of these diseases. Before the upper gastrointestinal endoscopic examination, height and weight were measured by physicians according to the standard protocol. Body mass index (BMI) was computed by dividing weight in kilograms by the square of height in meters and was stratified into categories, including underweight (BMI < 18.5 kg/m^2^), normal weight range (BMI 18.5–23.9 kg/m^2^), overweight (BMI 24–27.9 kg/m^2^), and obese (BMI ≥ 28 kg/m^2^).

### 2.4. EPL and ESCC Assessment

All eligible participants underwent an upper gastrointestinal endoscopic examination with Lugol’s iodine staining at baseline, under the supervision of a well-trained physician. After being given oral anesthesia, the esophagus of each participant was subjected to an application of 1.2% Lugol’s iodine solution. This particular solution imparts a brown hue to normal mucosal tissue, whilst dysplastic lesions are conspicuously devoid of any staining. A visual inspection of the entire esophagus was then conducted, and any suspicious esophageal lesions detected (non-stained regions with a diameter exceeding 5 mm) underwent further biopsy. Biopsy slides of esophageal tissue were independently read by two pathologists, with the quantity of biopsies being contingent upon the diameter of the lesion (1 biopsy for 5–19 mm, 2 biopsies for 20–39 mm, and 3 biopsies for ≥40 mm). Participants were classified as: (1) having normal pathology or esophagitis/basal cell hyperplasia; (2) EPLs (ESD as their histological criterion); and (3) ESCC, defined as carcinoma in situ, intramucosal carcinoma, and invasive carcinoma. The pathological criteria of determining EPL and ESCC outcomes were consistent with previous descriptions [23,29,30]. In the current analysis, we evaluated three EPL outcomes: overall EPLs that included all cases of mild, moderate, and severe dysplasia; EPLs with mild dysplasia only; and EPLs with high-grade dysplasia (moderate and severe dysplasia combined). We combined moderate and severe dysplasia, owing to the established association of high-grade dysplasia as a risk factor with a precancerous phase in the progression of esophageal squamous-cell carcinoma [31].

### 2.5. Statistical Analysis

We excluded 260 participants with missing diet data and 25 participants who reported an implausibly low or high total energy intake (<400 or >10,000 kcal/d) from the analyses [32]. The final analysis included 3967 residents. Participants were divided into tertiles of the DII score (tertile 1: −3.694 to 1.994; tertile 2, 1.994 to 2.944; tertile 3, 2.944 to 5.474). Baseline characteristics by tertile of DII score were presented by means and standard deviations for continuous variables and numbers and frequencies for categorical variables. Odds ratios (ORs) and 95% confidence intervals (CIs) of EPLs and ESCC were estimated using logistic regression with two different models. Model 1 was adjusted for age, sex, and total energy intake; Model 2 was further adjusted for marital status, educational level, occupation, BMI, household income, smoking status, alcohol drinking status, physical activity, and history of digestive diseases. These adjusted variables were identified as potential confounders in the DII and ESCC/EPL association based on the previous literature on this topic [33,34,35]. The linear trend in the odds of ESCC and EPLs across tertiles of DII scores was tested using a continuous DII score. The DII score was also analyzed as a continuous variable, with continuous ORs and 95% CIs estimated for each one-unit increase in the DII score after cubic spline analyses indicated the linear assumption was adequate [36].

Stratified analyses of the associations of the DII score with overall EPLs, EPLs with mild dysplasia, and ESCC were also conducted for variables selected a priori based on previous evidence of them being potential effect modifiers [33,34,35,37,38,39,40]. The potential effect modifiers evaluated included age, sex, BMI status, educational level, occupation, smoking status, alcohol drinking status, frequency of physical activity, history of digestive diseases, and eating habits (frequency of eating hot food and eating speed). We did not perform stratified analyses of EPLs with high-grade dysplasia due to very limited numbers of cases. In the stratified analyses of EPLs with mild dysplasia and ESCC, the DII score was modeled as a continuous variable to increase statistical power. Interactions between the DII score and each effect modifier were assessed by incorporating the cross-product term within the corresponding multivariable-adjusted logistic regression model. Due to the large number of effect modifiers, we further employed a Bonferroni corrected significance level of 0.005 (i.e., 0.05/11 = 0.0045) to account for multiple testing adjustments.

All analyses were executed utilizing SAS software (version 9.4, Cary, NC, USA). *p*-values less than 0.05 were regarded as indicative of statistical significance.

## 3. Results

### 3.1. Characteristics of Participants

**Figure 1:**
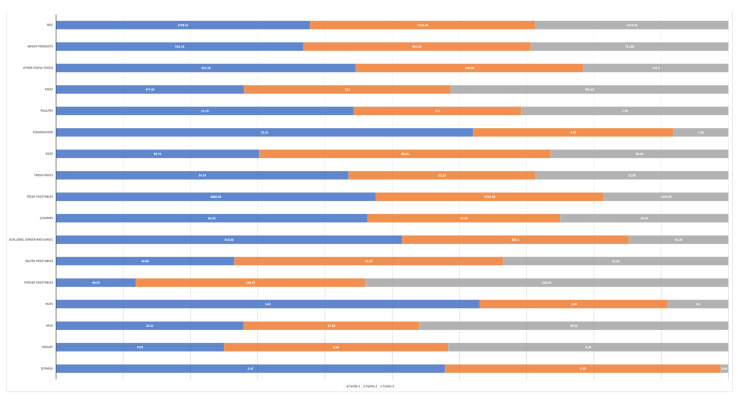
Means (grams/week) of 16 food groups consumed by 3967 participants by tertiles of DII scores in the NCEC-HRP study. Abbreviations: DII, Dietary Inflammatory Index; NCEC-HRP, The National Cohort of Esophageal Cancer-Prospective Cohort Study of Esophageal Cancer and Precancerous Lesions based on High-Risk Population.

The study population was comprised of 1993 (50.2%) male participants and 1974 (49.8%) female participants. The average age at study enrollment was 55.8 years. The mean (SD) DII score was 2.33 (3.55) and ranged from −3.69 to 5.47. Participants with higher DII scores tended to be older, female, farmers, non-smokers, non-drinkers, have lower educational level, lower household income, lower physical activity, poor or very poor self-rated health status and a history of digestive diseases. In terms of eating habits, they tended to eat hot food in the past year more frequently and were more likely to report a normal eating speed (Table 1). Participants with higher DII scores were more likely to consume less total energy, rice and wheat, fish or seafood, fresh vegetables and fruit, legumes, scallions, ginger and garlic; and more likely to consume more meat, milk and salted and pickled vegetables (Figure 1).

### 3.2. Associations of DII Scores with EPLs and ESCC

Upper gastrointestinal endoscopic examinations identified 312 participants with EPLs (*N* = 224 with mild dysplasia) and 72 with ESCC. A statistically significant positive association was shown between higher DII scores (i.e., a more pro-inflammatory diet) and odds of overall EPLs after adjusting for age, sex, and total energy intake (Model 1 OR_T3 vs. T1_ = 1.53, 95%CI = 1.05–2.23, *p*-trend = 0.05) (Table 2). The association was attenuated but still significantly positive in the fully adjusted model (Model 2 OR_T3 vs. T1_ = 1.45, 95%CI = 1.01–2.09). When modeled as a continuous variable, there were non-significant 12% higher odds of EPLs per one-unit increment in the DII score (Model 2 OR = 1.12, 95%CI = 0.96–1.31). A stronger association was observed with EPLs with high-grade dysplasia (OR_T3 vs. T1_ = 1.77, 95%CI = 0.86–3.61) than EPLs with mild dysplasia (OR_T3 vs. T1_ = 1.31, 95%CI = 0.83–2.06), although neither association was statistically significant. As for ESCC, participants consuming the most compared to the least pro-inflammatory diet had an 80% higher chance of developing ESCC (OR_T3 vs. T1_ = 1.80, 95%CI = 0.82–3.97), though this association was not statistically significant, likely due to the limited number of cases.

### 3.3. Stratified Analyses of Associations of DII Scores with EPLs and ESCC

**Figure 2:**
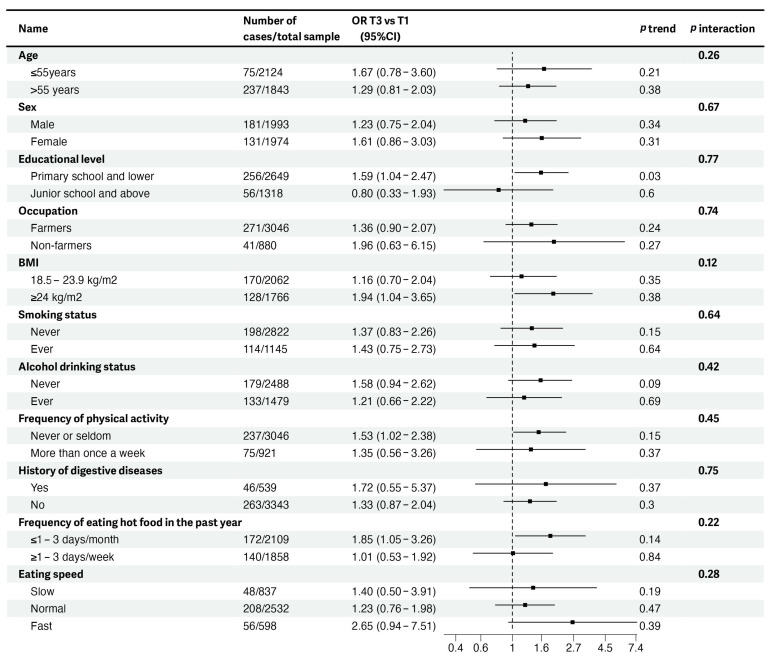
Stratified analyses of associations between DII scores (tertile 3 vs. tertile 1) and odds of overall EPLs in 3967 participants of the NCEC-HRP study. Model adjusted for age, sex, total energy intake, marital status, educational level, job, household income per year, BMI status, smoking status, alcohol drinking status, frequency of physical activity, and history of digestive diseases. The *p* value for trend was obtained from models with DII score as a continuous variable. The *p* value for interaction was calculated from the multivariable-adjusted model by adding the cross-product of DII score (continuous) and each effect modifier. The median age among 3967 participants was 55. The BMI category of “<18.5” was not present due to small sample size. Frequency of physical activity refers to occasions in an average week on which an individual’s heart rate obviously increased due to participation in physical activities. The category of “missing” in the digestive disease history variable was not present due to small sample size. Digestive diseases refer to superficial gastritis, atrophic gastritis, hypertrophic gastritis, stomach ulcer, and duodenal ulcer. Abbreviations: DII, Dietary Inflammatory Index; EPLs, esophageal precancerous lesions; NCEC-HRP, The National Cohort of Esophageal Cancer-Prospective Cohort Study of Esophageal Cancer and Precancerous Lesions based on High-Risk Population; OR, odds ratio.

We conducted subgroup analyses to further explore the associations between DII scores with overall EPLs, EPLs with mild dysplasia, and ESCC. Positive associations between DII scores and overall EPLs were only found in those whose educational level was below junior school (OR_T3 vs. T1_ = 1.59, 95%CI = 1.04–2.47), who were obese (OR_T3 vs. T1_ = 1.94, 95%CI = 1.04–3.65), and who ate hot food ≤1–3 days per month in the past year (OR_T3 vs. T1_ = 1.85, 95%CI = 1.05–3.26), but not in their counterparts (Figure 2). As for odds of mild EPLs, significantly positive associations with continuous DII score were also observed in the subgroup of participants with a lower educational level (OR = 1.23, 95%CI = 1.01–1.52) (Table S1). In subgroup analyses of DII and ESCC, significantly positive associations were identified among subjects with history of digestive diseases only (OR = 2.71, 95%CI = 1.01–7.73) (Table S2). However, none of these significant stratified associations reached the Bonferroni corrected significance level after multiple testing adjustments, and no significant interaction was found between DII scores and any effect modifier for any ESCC or EPL outcome.

## 4. Discussion

In this cross-sectional investigation of a Chinese cohort at high risk of ESCC, the consumption of a pro-inflammatory diet, as manifested in elevated DII scores, was observed to be significantly positively related to higher odds of EPLs compared to a diet with less inflammatory potential. The associations with mild and moderate/severe EPLs and ESCC were relatively similar in magnitude but not statistically significant, likely due to small case numbers. No statistically significant interactions were observed for any outcome, again likely due to limited statistical power.

In our study, individuals with higher DII scores consumed less energy but more pro-inflammatory foods such as meat, salted vegetables, and pickled vegetables (*p* < 0.01). It is noteworthy that salted or pickled foods are staple dietary items in Yanting because residents preserved foods with salt as a long local tradition, mainly due to the unavailability of refrigerators in these rural regions until the past decade [41]; however, salted and pickled foods have been found to significantly increase the risk of ESCC and induce inflammation, largely due to carcinogens such as *N*-nitroso compounds and heterocyclic amines formed through the preservation procedure [41,42,43]. In addition to salted and pickled foods, red meat consumption is another dietary factor that was associated with higher DII scores. There has been substantial evidence linking red meat, particularly processed red meat, to gastrointestinal (GI) cancers, including ESCC [44]. This may largely be due to the presence of potential carcinogens such as heme iron, polycyclic aromatic hydrocarbons, and heterocyclic amines, which are either naturally present or formed during cooking processes [45]. These compounds can induce oxidative stress and inflammation, thereby contributing to higher DII scores and, ultimately, the risk of ESCC. Conversely, fresh vegetables and fruits, rich in antioxidants, vitamins, and phytonutrients, generally have anti-inflammatory properties [46]. Their consumption has been inversely correlated with systemic inflammation [47]. The protective effect of a diet rich in vegetables and fruits on ESCC risk has also been highlighted [47]. In our study, higher DII scores were largely driven by the intake of pro-inflammatory foods, which suggested that in our study population, higher DII scores might be attributable to the consumption of foods with more pro-inflammatory potential rather than the large amount of foods consumed.

ESD is commonly observed in the esophageal mucosa and is the sole histopathological marker predictive of ESCC progression. As the grade of dysplasia increases, ESCC risk increases, as shown in the Linxian Dysplasia Nutrition Intervention Trial; compared to normal histology, the relative risk for ESCC was 3 for mild dysplasia, 10 for moderate dysplasia, and 30 for severe dysplasia [48]. Nonetheless, the etiological determinants of premalignant lesions in the squamous esophagus are largely unknown. Several possible risk factors for EPLs have been suggested, including elevated systolic blood pressure, a familial history of cancer, utilization of home heating devoid of a chimney, tooth loss, and higher concentrations of serum 25-hydroxyvitamin D [49,50,51]. With regards to dietary factors and EPL risk, a large cross-sectional study conducted in China which included 667 cases of EPLs found that and irregular diet (like skipping breakfast) and the consumption of corn flour, corn, pickled food, hot food, fried food, and liquor was associated with higher odds of EPLs, whereas the consumption of vegetables and fruits was associated with lower EPL odds [52]. Findings from this study are consistent with ours, as corn and corn flour, which contain a large amount of carbohydrates and fried food (mostly animal-based foods) are all pro-inflammatory within the context of DII [13,52]. We observed non-significant positive associations with mild and high-grade dysplasia, largely attributable to their small case numbers, calling for larger studies in the future to investigate how DII scores may impact the precancerous stages of ESCC.

We identified a positive though non-significant association between DII scores and ESCC, likely due to the limited number of ESCC cases. A meta-analysis including eight case-control studies concluded a 2.5-fold higher EC risk in the highest DII category compared with the lowest (95%CI = 1.90–3.40) [35]. Apart from DII scores, in prospective cohort studies, better adherence to other a priori dietary indices with strong anti-inflammatory potential, including the Mediterranean diet and the Healthy Eating Index-2005, was also found to be significantly inversely associated with ESCC risk [53,54]. Comparably, in another Chinese cross-sectional study carried out in five high-risk rural areas on 34,707 adults, a “healthy” dietary pattern rich in vegetables and fruits, ginger, and garlic was shown to be protective against ESCC, while a “Western” pattern characterized by higher levels of red meat and pickle consumption was associated with an increased risk of ESCC [55].

The positive association of DII scores with EPLs and ESCC prompted further subgroup analyses. However, after Bonferroni correction, these subgroup interactions were not statistically significant, consistent with findings in previous studies [39]. This suggests the intricate nature of such associations and highlights the potential need for replication studies with larger sample sizes. A notable consideration is the role of alcohol; while it is recognized in the DII for its anti-inflammatory potential, it is a known risk factor for EPLs and ESCC [56]. Upon metabolism, alcohol generates acetaldehyde, a detrimental compound. Acetaldehyde has been shown to induce DNA and protein damage, facilitating carcinogenic pathways [57,58]. This elucidates the intricate role of alcohol metabolic derivatives in enhancing cancer susceptibility, particularly within tissues with direct alcohol exposure such as the esophagus. Our study did not discern a significant interaction of DII with alcohol drinking status (never, ever), but there is potential for alcohol and dietary inflammation to jointly amplify ESCC risk. Existing research has indicated that large amounts of alcohol might intensify inflammation in the gastrointestinal tract [59]. Future studies with larger samples are essential to explore the interactions of DII scores with EPLs and ESCC.

The biological mechanisms explaining how a pro-inflammatory diet may lead to precancerous lesions and promote ESCC are not clear. One potential mechanism could involve a greater synthesis of pro-inflammatory cytokines such as vascular endothelial growth factor, CRP, and IL-8, which in turn may contribute to the formation of a microenvironment conducive to the development of ESCC. This may be achieved through an array of processes, including the stimulation of cellular proliferation and angiogenesis, and the curtailing of the mobilization of immune cells to the tumor locale [60,61,62]. Several dietary factors with low inflammatory potential, such as curcumin, fiber, and *n*-3 fatty acids, have been shown to activate anti-inflammatory signaling cascades, which have been implicated in the pathogenesis of esophageal cancer [39,63,64]. Another possible pathway might be through genetic mutations related to inflammation as ESD and ESCC samples have exhibited analogous mutations and markers of genomic instability. Further, the level of inflammation observed in these high-risk samples was correlated with atypical cell structures and indicators of DNA damage [65,66]. Molecular pathogeneses of the progression from squamous dysplasia to ESCC are not clear; therefore, further research is needed to elucidate the possible mechanisms through which diet may impact each stage in this progression.

In addition to dietary influences, the role of obesity in EPL and ESCC susceptibility cannot be understated. While our research adjusted for BMI, the intricate nexus between obesity and cancer pathogenesis requires more comprehensive elucidation. Epidemiological studies underscore the prominence of obesity as a significant risk determinant for esophageal malignancies [67]. The accumulation of adipose tissue, particularly visceral adiposity, induces a chronic inflammatory state [68]. This inflammatory milieu can instigate DNA aberrations, potentially triggering oncogenic transformations [69]. Given the burgeoning global obesity trajectory, it is imperative for subsequent research initiatives to intricately dissect the tripartite relationship between obesity, dietary patterns, and ESCC, aiming to delineate efficacious prophylactic interventions.

This study is among the first to explore the relation between the DII and precancerous-stage EPL as well as subtypes of EPLs of different severities to examine whether different stages of ESCC development might be related to diet-included inflammation. The ascertainment of EPL and ESCC cases through biopsy gave rise to the accurate classification of different EPLs, EPL stages, and ESCC. The validated FFQs captured foods and dietary habits local to Yanting. Participants were inquired about their food intake preceding the endoscopic examination when they were unaware of their disease status to avoid possible reverse causality, particularly for EPLs, which are generally asymptomatic. However, the study has several limitations, including its observational nature, which limited causality inference, potential measurement error in self-reported dietary data, and the small number of ESCC cases and EPL cases of each severity subtype, especially in stratified analyses, which may have affected the statistical significance of the results. Additionally, residual or unmeasured confounding factors may still exist. Nevertheless, given that EPLs, and notably ESD, predominantly manifest as asymptomatic [70], it is improbable for individuals to undertake dietary modifications consequent to EPLs. Consequently, the propensity for reverse causation, inherent to cross-sectional design, remains comparatively attenuated.

## 5. Conclusions

Consuming pro-inflammatory diets was associated with higher odds of EPLs and ESCC in a Chinese population at high risk of developing ESCC. Future prospective studies with large numbers of EPL and ESCC cases are warranted to evaluate further potential relationships between DII scores and EPLs, including subtypes of EPLs with different severity levels, and ESCC, and investigate whether these associations are modified by important lifestyle and clinical factors to guide efforts to prevent this highly fatal cancer.

## Figures and Tables

**Table 1:** Baseline characteristics of 3967 participants by Tertiles of DII score from diet in the NCEC-HRP Study.

Characteristics	DII Score		
	Tertile 1 (−3.694–1.994)	Tertile 2 (1.995–2.944)	Tertile 3 (2.945–5.474)
*N*	1322	1323	1322
	**Mean ± SD**	**Mean ± SD**	**Mean ± SD**
**Age (years)**	54.86 ± 7.77	56.37 ± 7.94	56.24 ± 7.84
**Total energy intake (kcal/day)**	1321.22 ± 220.38	1095.68 ± 179.01	896.78 ± 214.48
	**N (%) ^a^**	**N (%) ^a^**	**N (%) ^a^**
**Sex**			
Male	858 (64.9)	616 (46.6)	519 (39.3)
Female	464 (35.1)	707 (53.4)	803 (60.7)
**Marital status ^b^**			
Married	1269 (96.0)	1268 (95.8)	1281 (96.9)
Not married	53 (4.0)	55 (4.2)	41 (3.1)
**Educational level**			
Primary school and lower	794 (60.1)	920 (69.5)	935 (70.7)
Junior school	462 (35.0)	372 (28.1)	339 (25.6)
High school and above	66 (5.0)	31 (2.3)	48 (3.6)
**Occupation**			
Farmers	917 (69.4)	1096 (82.8)	1033 (78.1)
Non-farmers	405 (30.6)	227 (17.2)	289 (21.9)
**Household income (CNY/year)**			
<50,000	954 (72.2)	1152 (87.1)	1185 (89.6)
≥50,000	368 (27.8)	171 (12.9)	137 (10.4)
**BMI (kg/m^2^)**			
<18.5	15 (1.2)	39 (3.0)	45 (3.4)
18.5–23.9	640 (49.0)	721 (55.0)	701 (53.4)
24.0–27.9	502 (38.5)	459 (35.0)	477 (36.4)
≥28.0	148 (11.3)	91 (7.0)	89 (6.8)
**Smoking status**			
Never	804 (60.8)	973 (73.5)	1045 (79.1)
Past	74 (5.6)	80 (6.1)	64 (4.8)
Current	444 (33.6)	270 (20.4)	213 (16.1)
**Alcohol drinking status**			
Never	633 (47.9)	831 (62.8)	1024 (77.5)
Ever	689 (52.1)	492 (37.2)	298 (22.5)
**Frequency of physical activity ^c^**			
Never or seldom	921 (69.7)	941 (71.1)	1184 (89.6)
More than once a week	401 (30.3)	382 (28.9)	138 (10.4)
**Self-rated health status**			
Good	167 (12.6)	195 (14.8)	113 (8.5)
Fair	1111 (84.0)	1018 (77.0)	1085 (82.0)
Poor and very poor	44 (3.3)	109 (8.2)	125 (9.4)
**History of digestive diseases ^d^**			
Yes	162 (12.3)	163 (12.3)	260 (19.7)
No	1144 (86.5)	1148 (86.8)	1051 (79.5)
Missing	16 (1.2)	12 (0.9)	11 (0.8)
**Frequency of eating hot food in the past year ^e^**			
≤1–3 days/month	970 (73.4)	718 (54.3)	421 (31.8)
≥1–3 days/week	352 (26.6)	605 (45.7)	901 (68.2)
**Texture of staple food ^f^**			
Hard	118 (13.7)	105 (7.9)	132 (10.0)
Soft or liquid	1141 (86.3)	1218 (92.1)	1190 (90.0)
**Eating speed**			
Slow	337 (25.5)	260 (19.7)	240 (18.2)
Normal	759 (57.4)	863 (65.2)	910 (68.8)
Fast	226 (17.1)	200 (15.1)	172 (13.0)

Abbreviations: BMI, body mass index; DII, Dietary Inflammatory Index; NCEC-HRP, The National Cohort of Esophageal Cancer-Prospective Cohort Study of Esophageal Cancer and Precancerous Lesions based on High-Risk Population; SD, standard deviation. ^a^ Percentages for a categorical variable may not add up to 100% because of rounding or small number of missing variables. ^b^ “Not married” refers to single/divorced/widowed status. ^c^ Frequency of physical activity refers to occasions in an average week on which an individual’s heart rate obviously increased due to participation in physical activities. ^d^ Digestive diseases refers to superficial gastritis, atrophic gastritis, hypertrophic gastritis, stomach ulcer, and duodenal ulcer. ^e^ “Never”, “seldom” and “1–3 days/month” were merged into the categories of “≤1–3 days/month”; “1–3 days/week”, “4–6 days/week” and, “Everyday” were merged into the categories of “≥1–3 days/week”. ^f^ The related question asked in the interview was: “What is the texture of the staple food you usually consume?”.

**Table 2:** Associations between DII score and odds of EPLs and ESCC in 3967 participants of the NCEC-HRP study.

	Tertile 1	Tertile 2	Tertile 3	OR_continuous_^a^	*p*_trend_ ^b^
**Overall EPLs**					
Mean (range) ^c^	1.11 (−3.69–1.99)	2.45 (2.00–2.94)	3.44 (2.95–5.47)		
Cases/Total Sample	99/1322	95/1323	118/1322		
Model 1, OR (95%CI) ^d^	Ref.	0.98 (0.71–1.35)	1.53 (1.05–2.23)	1.15 (1.00–1.34)	0.05
Model 2, OR (95%CI) ^e^	Ref.	0.94 (0.68–1.31)	1.45 (1.01–2.09)	1.12 (0.96–1.31)	0.15
**Mild EPLs**					
Mean (range) ^c^	1.10 (−3.69–1.99)	2.45 (2.00–2.94)	3.44 (2.95–5.47)		
Cases/Total Sample	76/1299	69/1297	79/1283		
Model 1, OR (95%CI) ^d^	Ref.	0.95 (0.66–1.38)	1.39 (0.90–2.16)	1.14 (0.96–1.36)	0.14
Model 2, OR (95%CI) ^e^	Ref.	0.94 (0.65–1.37)	1.31 (0.83–2.06)	1.11 (0.93–1.34)	0.21
**Moderate and Severe EPLs**					
Mean (range) ^c^	1.10 (−3.69–1.99)	2.45 (2.00–2.94)	3.44 (2.95–5.47)		
Cases/Total Sample	23/1245	26/1254	39/1242		
Model 1, OR (95%CI) ^d^	Ref.	1.07 (0.58–1.97)	1.97 (0.99–3.92)	1.18 (0.92–1.53)	0.20
Model 2, OR (95%CI) ^e^	Ref.	0.96 (0.52–1.79)	1.77 (0.86–3.61)	1.15 (0.87–1.51)	0.33
**ESCC**					
Mean (range) ^c^	1.11 (−3.69–1.99)	2.45 (2.00–2.94)	3.44 (2.95–5.47)		
Cases/Total Sample	19/1322	21/1323	32/1322		
Model 1, OR (95%CI) ^d^	Ref.	1.08 (0.55–2.11)	1.93 (0.90–4.11)	1.13 (0.85–1.49)	0.40
Model 2, OR (95%CI) ^e^	Ref.	0.96 (0.48–1.92)	1.80 (0.82–3.97)	1.14 (0.84–1.55)	0.39

Abbreviations: DII: Dietary Inflammatory Index; ESCC: esophageal squamous-cell cancer; EPLs: esophageal precancerous lesions; NCEC-HRP, The National Cohort of Esophageal Cancer-Prospective Cohort Study of Esophageal Cancer and Precancerous Lesions based on High-Risk Population; OR, odds ratio. ^a^ OR_continuous_ was calculated per one-unit increase in DII score. ^b^ *p* value for trend was obtained from models with DII score as a continuous variable. ^c^. Mean value here refers to mean DII score of total samples (cases & non-cases) in the category. ^d^ Model adjusted for age (continuous), sex (male, female), and total energy intake (continuous). ^e^ Model further adjusted for marital status (married, not married), educational level (primary school and lower, junior school, high school and above), occupation (farmers, non-farmers), household income (<CNY 50,000, ≥CNY 50,000/year), BMI (<18.5, 18.5–23.9, 24–27.9, ≥28 kg/m^2^), smoking status (never, past, current), alcohol drinking status (never, ever), frequency of physical activity (never or seldom, more than once a week), history of digestive diseases (yes, no).

## Data Availability

The data presented in this study are available on request from the corresponding author. The data are not publicly available.

## Supplementary Materials

The following supporting information can be downloaded at: https://www.mdpi.com/article/10.3390/nu15184078/s1: Table S1: Stratified analyses of the associations between DII scores and odds of mild EPL in 3967 participants in the NCEC-HRP study; Table S2: Stratified analyses of the associations between DII scores and odds of ESCC in 3967 participants in the NCEC-HRP study.
