# Supplementary material for: Associations of Dietary Inflammatory Potential with Esophageal Precancerous Lesions and Esophageal Squamous-Cell Cancer: A Cross-Sectional Study
Source: Nutrients. 2023 Sep 21;15(18):4078. doi: 10.3390/nu15184078 (PMC10537352; doi:10.3390/nu15184078)
Supplement: Supplementary file 1 [file nutrients-15-04078-s001.zip › Table S1.pdf]

Supplemental Table S1. Stratified analyses of the associations between DII scores from diet and odds of mild EPL in 3,967 participants, the NCEC-HRP Study [a]

	Number of cases / total sample	*OR continuous* [b]	*P value* [c]	$P_{interaction}$ [d]
**Age [e]**				0.20
≤55 years	62/2124	1.27 (0.91-1.78)	0.15	
>55 years	164/1843	1.06 (0.85-1.31)	0.62	
**Sex**				0.29
Male	126/1993	1.06 (0.85-1.33)	0.59	
Female	100/1974	1.22 (0.88-1.69)	0.24	
**Educational level**				0.54
Primary school and lower	183/2649	1.23 (1.01-1.52)	0.04	
Junior school and above	43/1318	0.79 (0.54-1.16)	0.23	
**Job**				0.74
Farmers	191/3046	1.10 (0.91-1.34)	0.33	
Non-farmers	35/880	1.30 (0.77-2.21)	0.33	
**BMI status [g]**				0.17
18.5-23.9	123/2062	1.08 (0.85-1.37)	0.52	
⩾24	89/1766	1.17 (0.89-1.55)	0.27	
**Smoking status**				0.06
Never	146/2822	1.23 (0.97-1.56)	0.09	
Ever	80/1145	0.97 (0.73-1.29)	0.81	
**Alcohol drinking status**				0.21
Never	137/2488	1.24 (0.98-1.59)	0.08	
Ever	89/1479	1.00 (0.76-1.33)	0.98	
**Frequency of physical activity [h]**				0.14
Never or seldom	174/3046	1.17 (0.96-1.44)	0.12	
More than once a week	52/921	0.98 (0.66-1.47)	0.93	
**History of digestive diseases [i]**				0.89
Yes	34/539	1.13 (0.66-1.91)	0.66	
No	190/3343	1.09 (0.89-1.32)	0.40	
**Frequency of eating hot food in the past year**				0.26
⩽1-3 days/month	115/2109	1.20 (0.94-1.54)	0.14	
⩾1-3 days/week	109/1858	0.98 (0.73-1.31)	0.89	
**Eating speed**				0.58
Slow	37/837	1.12 (0.73-1.73)	0.60	
Normal	150/2532	1.05 (0.84-1.32)	0.65	
Fast	37/598	1.36 (0.88-2.11)	0.16	

Abbreviations: DII: Dietary Inflammatory Index; EPL: Esophageal Precancerous Lesions; NCEC-HRP, The National Cohort of Esophageal Cancer-Prospective Cohort Study of Esophageal Cancer and Precancerous

Lesions based on High-Risk Population; OR, odds ratio.

a. Model adjusted for age, sex, total energy intake, marital status, educational level, job, household income per year, BMI status, smoking status, alcohol drinking status, frequency of physical activity, history of digestive diseases.

b. OR $_{continuous}$ was calculated for one unit increase in DII score in association with disease risk.

c. The *P* values were obtained from models with DII score as a continuous variable.

d. The *P* value for interaction was calculated from the multivariable-adjusted model by adding the cross-product of DII score (continuous) and each effect modifier.

e. The median of age among 3967 participants was 55.

f. "Not married" refers to single/divorced/widowed status.

g. The category of "<18.5" was not presented due to small sample size.

h. Frequency of physical activity refers to the occasions in an average week that an individual's heart rate obviously increases due to participation in physical activities.

i. The category of "Missing" was not presented due to small sample size. Digestive diseases refer to superficial gastritis, atrophic gastritis, hypertrophic gastritis, stomach ulcer, and duodenal ulcer.
